# The importance of hypoglycemia in diabetic patients

**DOI:** 10.1186/2251-6581-11-17

**Published:** 2012-10-01

**Authors:** Gita Shafiee, Mohammadreza Mohajeri-Tehrani, Mohammad Pajouhi, Bagher Larijani

**Affiliations:** 1 Endocrinology and Metabolism Research Center, Tehran University of Medical Sciences, Shariati Hospital, North Kargar Street, 14114, Tehran, Iran

**Keywords:** Diabetes, Hypoglycemia, Complications

## Abstract

Hypoglycemia is one of the most important complications of diabetes treatment. The risk of severe hypoglycemia is higher in elderly patients, those having comorbidities such as vascular disease or renal failure, pregnant women and in children with type 1diabetes. Moreover, in type 2 diabetes, progressive insulin deficiency, longer duration of diabetes, and tight glycemic control increase the risk of hypoglycemia as much as type 1 diabetes.Episodes hypoglycemia may lead to impairment of counter-regulatory system, with the potential of development of hypoglycemia unawareness. So, hypoglycemia may increase the vascular events even death in addition to other possible detrimental effects. Glycemic control should be individualized based on patient characteristics with some degree of safety. Recognition of hypoglycemia risk factors, blood glucose monitoring, selection of appropriate regimens and educational programs for healthcare professionals and patients with diabetes are the major issues to maintain good glycemic control, minimize the risk of hypoglycemia, and prevent long- term complications.

## Introduction

Diabetes is a chronic disease that requires continuous medical care and patient self-management education to prevent acute complications and reduce the risk of long-term complications [1]. The prevalence of diabetes has reached epidemic proportions in most populations. According to the UN World Health Organization (WHO) more than 220 million people worldwide have diabetes, from which more than 70% live in low- and middle income countries. It is expected that the number of diabetic subjects grows to 366 million by 2030, a figure that is more than twice the number in 2000. Epidemiologic evidences suggest that unless effective preventive measures are implemented the global prevalence will continue to rise [2].

In Middle East, internationally published data on national estimation of type 2 diabetes prevalence are spare. In Iran a prevalence of 7.7% in people younger than 65 years is reported [3]. Diabetes and its complications place a very heavy burden on health care systems. A study in our country showed that the burden of diabetes and its complications in term of Disability-Adjusted Life Years (DALYs) was 306,440 years [4]. All these points stress that prevention of this disease and its complications is important in Iran.

The overall objective of type 2 diabetes’s management is to achieve and maintain blood glucose control and reduce the risk of long-term complications. Many studies have shown that modern management with intensive glycemic control can limit, delayed or even prevent the chronic complications of diabetes. However this intensive diabetes treatment could be associated with an increased risk of hypoglycemia [5], particularly in patients with type 1 diabetes mellitus and patients with longstanding insulin-treated type 2 diabetes mellitus [6-9].

Hypoglycemia is a true medical emergency which requires prompt recognition and treatment to prevent organ and brain damage. The spectrum of symptoms depends on duration and severity of hypoglycemia and varied from autonomic activation to behavioral changes to altered cognitive function to seizures or coma. The short and long term complications include neurologic damage, trauma, cardiovascular events and death [10]. Severe untreated hypoglycemia can cause a significant economic and personal burden, therefore identification and prevention of hypoglycemia can reduce diabetes burden by prevention of hypoglycemia complications.

In this review we will give an overview of the current literature on impact of hypoglycemia on diabetes management and try to provide guidance to health care providers to safely and effectively assist the diabetic patients.

### Epidemiology

For diabetic patients hypoglycemia is a fact of life. Approximately 90% of all patients who receive insulin have experienced hypoglycemic episodes. The reported incidence of hypoglycemia varies considerably among studies, however in general patients with type1 diabetes have an average of two episodes of symptomatic hypoglycemia per week and one episode of severe hypoglycemia once a year. An estimated 2–4% of deaths of this population have been attributed to hypoglycemia [11,12].

It should be noted that the incidence of reported hypoglycemia is usually under- estimates because of difficulty to determine. The detection of hypoglycemia would require continuous blood glucose measurements. In this regard asymptomatic episodes of hypoglycemia could be missed unless detected by monitoring of blood glucose. On the other hand, the incidence of symptomatic episodes is difficult to determine because they are rarely reported. Additionally in some clinical trials hypoglycemia is not a primary outcome which makes it difficult to estimate the incidence of hypoglycemia. While the episodes of severe hypoglycemia are a small fraction of the total hypoglycemic, estimation of this event is the most reliable since severe hypoglycemia events are better documented [13]. The frequency of hypoglycemia is lower in people with type 2 diabetes than Type 1 [12]. The UK Hypoglycemia Study showed that in patients with type 2 diabetes the risk of severe hypoglycemia is low in the first few years (7%) and that risk increases to 25% later in the course of diabetes [14]. However the prevalence of type 2 diabetes is about twenty fold higher than type 1 diabetes and many patients with type2 diabetes finally require treatment with insulin, therefore most episodes of hypoglycemia occur in patients with type 2 diabetes.

The incidence of hypoglycemia also could be affected by how tight glycemic control is performed. The Action to Control Cardiovascular Risk in Diabetes (ACCORD) reported a threefold increase in severe hypoglycemia and coma in intensively treated patients versus conventionally treated patients [15].

### Pathophysiology

Glucose is an obligate metabolic fuel for the brain under physiological conditions. Because the brain cannot synthesize glucose, maintenance of brain function requires a virtually continuous supply of glucose from the circulation. In normal situation redundant glucose counter-regulatory mechanisms effectively prevent or rapidly correct hypoglycemia [13].

When hypoglycemia happens, a decrease in insulin secretion is the first response. Other hormones including glucagon and epinephrine are also secreted promptly after falling plasma glucose levels and both induce a rapid increase in glucose production [16].

Hypoglycemia typically arises when abnormalities in the mechanisms involved in glucose homeostasis is existed. In patients with type 1 diabetes secretion of both counter-regulatory hormones of insulin and glucagon is severely interrupted. The third defense mechanism, the epinephrine response to hypoglycemia, is also become progressively impaired in type1 diabetic patients [17]. The frequent bouts of hypoglycemia further reduce the sympathoadrenal glycemic threshold to a lower plasma glucose level particularly in patients with an intensive regulation of diabetes [18]. The combination of glucagon absence and the attenuated epinephrine response causes the clinical syndrome of defective glucose counter- regulation, a syndrome that has been shown to increase the risk of severe hypoglycemia by 25- fold or even higher during strict treatment compared to when a normal epinephrine responses is presented [19]. Decreased in epinephrine response to hypoglycemia is a marker of an attenuated autonomic neural response that causes the clinical syndrome of hypoglycemia unawareness, [20], a situation that in turn could increases the risk of severe hypoglycemia development.

Hypoglycemia-associated autonomic failure (HAAF) in type1 diabetes apparently results from recent antecedent hypoglycemia that caused by both defective counter-regulatory response and hypoglycemia unawareness. Thus hypoglycemia caused HAAF may play a role in the vicious cycle of recurrent hypoglycemia [12,21].

The mechanisms of defect in glucose counter-regulation in type 2 diabetes are the same as type 1diabetes which includes both defect in hypoglycemia counter-regulation mechanism and deficiency of sense of hypoglycemia [6]. Similar to type 1 diabetes intensive therapy could lead to development of defective counter-regulatory responses. A study revealed that individuals with type 2 diabetes develop defects in counter-regulatory responses to hypoglycemia after exposure to a single episode of hypoglycemia. These patients can also develop blunted physiological defenses to hypoglycemia following intensive oral therapy. This suggests that people with type 2 diabetes who are strictly treated are more likely to develop impaired hypoglycemia awareness [22].

### Causes and risk factors for hypoglycemia

In general, hypoglycemia in diabetic patients occurs when an imbalance between insulin/hypoglycemic agent’s intake and body^’^s physiological need exists. The following reasons could account for hypoglycemia in diabetics: Iatrogenic, Diet changes and infections.

Diabetes medications including insulin and sulphonylureas are among the most common causes of hypoglycemia in diabetic subjects [23]. The longer-acting sulphonylureas such as glibenclamide and chlorpropamide are associated with more severe hypoglycemia than the shorter-acting drugs [24]. Occasional episodes of hypoglycemia with Metformin, as the most commonly used anti-diabetic drug, are reported when an imbalance between food intake and dose of Metformin is presented [25].

Hypoglycemia may also result from little foods intake or increase in activity in relation to medication and food intake. Other causes such as alcohol consumption, some drugs, stress and infections should also be considered in diabetic subjects. Alcohol may contribute to the severity of hypoglycemia by inhibiting gluconeogenesis.

Hypoglycemia could also be a symptom of severe organ illnesses. Liver diseases such as hepatitis and cirrhosis as well as kidney diseases often cause hypoglycemia, mostly because of the major role that these organs play in glucose production and maintenance blood sugar levels.

Hypoglycemia unawareness and a history of previous severe hypoglycemia episodes are risk factors for severe hypoglycemia. Impaired symptomatic awareness is associated with a 6-fold and 9-fold increased risk of severe hypoglycemia in patients with type1 and type 2 diabetes respectively [8,20]. Patients receiving intensive insulin therapy are at increased risk of hypoglycemia despite lack of other risk factors.

In type 2 diabetes progressive insulin deficiency and duration of insulin therapy increase the risk of hypoglycemia as in type 1 diabetes, and the risk of hypoglycemia is highest in those with type 2 diabetes who have received insulin for more than 10 years [7]. Moreover risk of severe hypoglycemia is higher in elderly patients, those with co-morbidities such as vascular disease or renal failure, pregnant women and in children with type 1diabetes [7,26].

#### Hypoglycemia and pregnancy

Pregnancy is associated with a high risk of severe hypoglycemia in diabetic subjects. Hypoglycemia in pregnant women may lead to severe morbidity and even death [27]. In women with diabetes severe hypoglycemia episodes occur three to five times more frequently in first trimester than last trimester [28,29].

Although intensive metabolic control can prevent diabetic complications in pregnant woman and her offspring, it may increase the risk of hypoglycemia [28]. History of previous hypoglycemia in the year preceding pregnancy, hypoglycemia unawareness, long duration of diabetes, and fluctuation in glucose levels, have been documented as risk factors of severe hypoglycemia during pregnancy [28-30]. Pregnancy itself is associated with suppression of glucose counter- regulatory responses. The exact mechanism for this suppression is unclear; however some studies have shown that reduced sympathoadrenal responses during hypoglycemia may contribute to defective glucose counter-regulation and impaired awareness of hypoglycemia [31,32].

Tight metabolic control with focus on reduction of glucose fluctuations during pregnancy improves the pregnancy outcome. Appropriate self-management training including carbohydrate counting, hypoglycemia awareness, and clear insulin dose adjustment instruction, are important in diabetic-pregnant women in order to maintain near-normoglycemic state without episodes of severe hypoglycemia particularly in early pregnancy stage.

#### Hypoglycemia in elderly

Hypoglycemia is a common problem in old people with diabetes. Aging modifies the cognitive, symptomatic, and counter-regulatory hormonal responses to hypoglycemia [33]. The effect of aging on increased risk of unawareness or severe episodes of hypoglycemia has also been recognized [34]. Although hypoglycemia in the elderly is the most common complication of tight glycemic control, multiple co-morbidities like renal impairment, chronic heart disease, malnutrition and polypharmacy may increase risk of this complication [34].

In the elderly subjects, episodes of hypoglycemia are more likely to be followed by changes in the blood brain circulation which may further increase the risk of neurological damage in this population [35,36].

Severe hypoglycemia has a considerable impact on wellbeing, productivity and quality of life in old people with diabetes [37]. Management goals for elderly patients should be an individualized process and must include a number of considerations. Numerous studies have demonstrated the benefits of avoiding intensive attenuation of HbA1C to prevent severe hypoglycemia. Patient training and treatment of the early symptoms of hypoglycemia may prevent the occurrence of further severe hypoglycemia and decrease the rate of hospitalization, mortality and cognitive impairment that directly affects the independence and functionality of older persons.

#### Hypoglycemia in children and adolescents

Hypoglycemia is one of the most common acute complications of insulin therapy in children and adolescents with diabetes. The incidence of hypoglycemia is reported to be between 3 and 27 episodes per 100 patient- year in children with type 1diabetes [38]. The recurrent and severe episodes of hypoglycemia cause hypoglycemic fearfulness and emotional morbidity both for patients and their parents, which could act as a limiting factor in achievement of good glycemic control.

Impaired hypoglycemia awareness is a significant problem for children with type 1diabetes and a major risk factor for development of severe hypoglycemia [39]. Children with early-onset of diabetes, particularly those diagnosed before age of 6, and severe episodes of hypoglycemia have an increased range of cognitive dysfunction and brain abnormalities [40]. Repeated hypoglycemic seizures in young children may also cause structural brain changes [41].

Therefore regular glucose monitoring and detection of the risk factors of hypoglycemia may help to reduce the severity and frequency of hypoglycemia in children and adolescent with diabetes.

### Impact of hypoglycemia

Hypoglycemia can cause severe morbidity and even death, usually depending on its severity or duration.

#### Hypoglycemia and vascular disease

A possible link between hypoglycemia and acute vascular events like angina, myocardial infarction, and acute cerebrovascular disease has been proposed by several studies [42,43]. In addition some studies have highlighted an increased risk of death and microvascular complications when glucose levels are intensively controlled [15,44].

However some controversies exist in this issue. The Action to Control Cardiovascular Risk in Diabetes (ACCORD) trial could not establish the direct role of hypoglycemia in increased cardiovascular events [15], while a much stronger link between severe hypoglycemia and vascular events is indicated in the Veteran’s Affairs Diabetes Trial (VADT) study [45]. Gimenez *et al.*[46] suggested that repeated episodes of hypoglycemia may promote the development of macrovascular disease in type 1diabetes by increasing the risk of atherosclerosis. Sympathoadrenal stimulation and release of counter-regulatory hormones during acute hypoglycemia causes hemodynamic changes including white blood cell activation and inflammatory mediators and cytokines release [47,48]. Changes in regional blood flow, localized vasoconstriction, an increased risk of intravascular coagulation and endothelial damage, could provoke tissue ischemia [49]. While chronic hyperglycemia is probably the main driver of preclinical atherosclerosis in diabetes [50], recurrent exposure to hypoglycemia may contribute to this process by aggravating established micro and macrovascular complications [51].

In addition hypoglycemia could potentially increase the sudden death through inducing either ischemic or other electrocardiographic changes (increased QTc), which in turn may predispose the diabetic subjects to ventricular arrhythmias [45,52].

#### Hypoglycemia and the brain

A new research suggests a possible association between severe hypoglycemia and cognitive dysfunction. Åsvold et al*.*[53] suggested that early exposure to severe hypoglycemia could have clinically relevant effects on cognition many years later. In this study the overall cognitive scores of the 9 diabetic children who experienced severe hypoglycemia before age of ten were lower than the eighteen diabetic children without history of severe hypoglycemia. The same result was evident when all children were re-examined as adults 16 years later.

Many studies showed that the development of diabetes in the first stages of life is associated with an increased risk of neurocognitive dysfunction regardless of whether or not severe hypoglycemia was present [54]. It has been hypothesized that the diabetes-associated metabolic changes may disrupt the process of normal brain development in the first years of life. Severe hypoglycemia may aggravate the severity of the brain dysfunction in people with an early onset of diabetes [55,56]. Gaudieri et al*.* found that risk factors for cognitive impairment in children with diabetes include: hypoglycemia, duration of diabetes and poor glycemic control [57].

In type 2 diabetes, one longitudinal cohort study in elderly patients revealed that severe hypoglycemia episodes are associated with an increased risk of dementia in this population, although the impact of mild episodes on dementia risk remains unknown [58].

A recent study found that severe hypoglycemia causes brain damage in cortex and the hippocampus regions and the extent of damage was closely correlated to the presence of seizure-like activity. The results were indicative of elevation of sensitivity of the cortex to the damaging effects following an episode of severe hypoglycemia [35].

These researches remind us to be particularly alert for the child or adult who experience severe hypoglycemia early in life. This population is more likely to manifest developmental delays and cognitive dysfunction [54]. Their reports of poor performance in school, or difficulties at work may reflect CNS anomalies secondary to diabetes and its treatment, and may be agreeable to various remediation strategies [59].

#### Hypoglycemia and quality of life

Studies reported that severe hypoglycemia can have a significant impact on patients' health-related quality of life, treatment satisfaction, and cost of diabetic management. The wellbeing of patients may be affected both directly from the effects of hypoglycemia and indirectly from fear of recurrence [60,61]. Marret *et al.* found the positive association between severity/ frequency of hypoglycemic episode and greater fear of hypoglycemic events [62]. As a result hypoglycemic fearness makes the patients to modify their behaviors in a way to have less episodes of hypoglycemia, which this in turn could contribute to a negative glycemic control. Taken together these effects may lead to considerable increase in burden as well as attenuation of life quality.

### Prevention of hypoglycemia

While achieving and maintaining the optimal glycemic control is one of the principal aims of prevention and management of diabetes complications, hypoglycemia remains a major challenge [63]. Obviously prevention of hypoglycemia is preferable to its treatment since as compared with a reactive approach, prevention is much more likely to avoid severe events and economic burden. The prevention of hypoglycemia requires some principles consideration. These principles include: 1) diabetes self-management (supported by education and empowerment); 2) self- monitoring of blood glucose or continuous glucose sensing; 3) flexible and appropriate insulin or other drug regimens; 4) individualized glycemic goals; 5) consideration of known risk factors of hypoglycemia; 6) professional support and guidance [64,65].

Diabetes self-management, supported by education and empowerment, is a basic part of diabetes care to achieve successful health-related outcomes [66,67]. Several studies have found that diabetes self-management education (DSME) results in behavior changes with positive influence on outcome [68,69]. Patients with diabetes need to be well informed about the symptoms of hypoglycemia, to know about hypoglycemia risk factors, prevention and treatment, and to be concern about monitoring of blood glucose levels. Therefore educating the patients of all ages and their relatives about hypoglycemia is a key factor in prevention of this complication.

In addition, blood glucose monitoring (BGM), using any of the widely available self- monitoring blood glucose (SMBG) or interstitial glucose sampling using continuous glucose monitors (CGM); is an important part of management of diabetes; especially for people who experience hypoglycemic episodes [70]. BGM provides an immediate evaluation of blood glucose levels; information that can be used to guide the therapy and to detect the hypoglycemia, and offers important feedback both to patients and to the health cares about glycemic control and patient treatment satisfaction [71]. CGM may be particularly important for patients with hypoglycemia unawareness and/or patients experiencing frequent episodes of hypoglycemia [72].

It is important that in patients with a history of recurrent hypoglycemia, the time of episodes be identified and the treatment regimen be adjusted accordingly [65]. With a basal-bolus insulin regimen, morning fasting hypoglycemia may be caused by the long- or intermediate- acting insulin; daytime hypoglycemia is implicated by rapid or short acting insulin; and nocturnal hypoglycemia may be implicated by either. Substitution of short-acting (regular) insulin with rapid-acting insulin (e.g. lispro or aspart) reduces frequency of daytime hypoglycemia. Substitution of long-acting (e.g., glargine or determir) insulin for intermediate- acting insulin (e.g., NPH or premix 70/30) reduces frequency of nocturnal and daytime hypoglycemia [65,73].

Continuous subcutaneous insulin infusion (CSII) with a rapid-acting insulin analog improves the glycemic control and reduces the rate of hypoglycemia compared with multiple daily insulin injection [74].

Patients on oral anti-diabetic drugs are also at risk for developing hypoglycemia. Agents such as metformine, dipeptidyl peptidase-4 inhibitors, and thiazolidines are preferable to sulfonylureas in minimizing hypoglycemic risks [13].

Glycemic goals should be individualized with some degree of safety particularly for patients with long duration of diabetes; patients who have a high risk of severe hypoglycemia development, and/or subjects with multiple co-morbidities [11,75,76]. Individualized treatment should be determined by a close working relationship between diabetes care team and the patients. The health care professionals may improve patient knowledge and produce positive changes in life style and self-care decisions. The diabetes care team involvement not only provides the initial patient’s guidance, but also monitors the short and long term complications for early detection and management [77,78].

## Conclusions

Hypoglycemia is a major limiting factor in tight glycemic management of diabetes and may increase vascular events in addition to other possible detrimental effects. Glycemic control should be individualized based on patient characteristics with some degree of safety. Recognition of hypoglycemia risk factors, blood glucose monitoring, selection of appropriate regimens, education programs for healthcare professionals and patients with diabetes are the major issues to maintain good glycemic control, minimize the risk of hypoglycemia, and prevent long- term complications.

## Competing interests

The authors declare they have no competing interests.

## Authors' contributions

Dr. Shafiee participated in the drafting of the manuscript, Dr. Mohajeri-Tehrani participated in the study design, Dr. Pajouhi critically revised the manuscript, and Dr. Larijani was main supporter of the study and contributed in the writing of specific sections of the manuscript. All authors read and approved the final manuscript.
